# Exercise and postprandial lipemia: effects on vascular health in inactive adults

**DOI:** 10.1186/s12944-018-0719-3

**Published:** 2018-04-03

**Authors:** Robinson Ramírez-Vélez, María Correa-Rodríguez, Alejandra Tordecilla-Sanders, Viviana Aya-Aldana, Mikel Izquierdo, Jorge Enrique Correa-Bautista, Cristian Álvarez, Antonio Garcia-Hermoso

**Affiliations:** 1 Centro de Estudios en Medición de la Actividad Física (CEMA), Escuela de Medicina y Ciencias de la Salud, Universidad del Rosario, Bogotá, D.C, Colombia; 2 Faculty of Health Sciences, University of Granada, Granada, Spain; 3 Department of Health Sciences, Public University of Navarre, CIBER de Fragilidad y Envejecimiento Saludable (CB16/10/00315), Tudela, Navarre Spain; 4 Department of Physical Activity Sciences, Universidad de Los Lagos, Osorno, Chile; 5 Research Nucleus in Health, Physical Activity and Sports, Universidad de Los Lagos, Osorno, Chile; 6 Laboratorio de Ciencias de la Actividad Física, el Deporte y la Salud, Facultad de Ciencias Médicas, Universidad de Santiago de Chile, USACH, Santiago, Chile

**Keywords:** Postprandial lipemia, Endothelial function, Exercise intensity, High-intensity exercise, Moderate continuous training

## Abstract

**Background:**

There is evidence to suggest that postprandial lipemia are is linked to the impairment of endothelial function, which is characterized by an imbalance between the actions of vasodilators and vasoconstrictors. The aim of this study was to determine the effects of a 12-week high-intensity training (HIT) and moderate continuous training (MCT) protocol on postprandial lipemia, vascular function and arterial stiffness in inactive adults after high-fat meal (HFM) ingestion.

**Methods:**

A randomized clinical trial was conducted in 20 healthy, inactive adults (31.6 ± 7.1 years). Participants followed the two exercise protocols for 12 weeks. To induce a state of postprandial lipemia (PPL), all subjects received a HFM. Endothelial function was measured using flow-mediated vasodilation (FMD), normalized brachial artery FMD (nFMD), aortic pulse wave velocity (PWV) and augmentation index (AIx). Plasma total cholesterol, high-density lipoprotein cholesterol (HDL-c), triglycerides and glucose were also measured.

**Results:**

The effects of a HFM were evaluated in a fasted state and 60, 120, 180, and 240 min postprandially. A significant decrease in serum glucose between 0 min (fasted state) and 120 min postprandially was found in the HIT group (*P* = 0.035). Likewise, FMD (%) was significantly different between the fasted state and 60 min after a HFM in the HIT group (*P* = 0.042). The total cholesterol response expressed as area under curve (AUC)_(0–240)_ was lower following HIT than following MCT, but no significant differences were observed (8%, *P* > 0.05). Similarly, triglycerides AUC_(0–240)_ was also lower after HIT compared with MCT, which trended towards significance (24%, *P* = 0.076). The AUC_(0–240)_ for the glucose response was significantly lower following HIT than MCT (10%, *P* = 0.008). FMD and nFMD AUC_(0–240)_ were significantly higher following HIT than following MCT (46.9%, *P* = 0.021 and 67.3%, *P* = 0.009, respectively). PWV AUC_(0–240)_ did not differ following between the two exercise groups (2.3%, P > 0.05).

**Conclusions:**

Supervised exercise training mitigates endothelial dysfunction and glucose response induced by PPL. Exercise intensity plays an important role in these protective effects, and medium-term HIT may be more effective than MCT in reducing postprandial glucose levels and attenuating vascular impairment.

**Trial registration:**

ClinicalTrials.gov ID: NCT02738385 Date of registration: April 14, 2016.

**Electronic supplementary material:**

The online version of this article (10.1186/s12944-018-0719-3) contains supplementary material, which is available to authorized users.

## Background

Postprandial lipemia (PPL) is defined as the elevation of circulating triglyceride-rich lipoproteins after high-fat meal (HFM) consumption. There is evidence to suggest that these exaggerated elevations in triglycerides are linked to impairment of endothelial function, characterized by an imbalance between the actions of vasodilators and vasoconstrictors [1]. Although the pathophysiology of endothelial dysfunction has not been fully clarified, reduced nitric oxide (NO) and increased oxidative stress are important contributors to the reduction of the vasodilatory response [2].

Endothelial dysfunction induced by PPL is considered an early and reversible predictor of atherosclerotic disease and cardiac events [3, 4]. As humans spend a considerable part of the day in a postprandial state, interventions that can reduce the magnitude and duration of this metabolic state may be beneficial in the prevention of cardiovascular disease (CVD).

Exercise training prior to high-fat meal ingestion has been shown to have an attenuating effect on postprandial metabolism [5, 6]. In the same line, studies have reported that energy expenditure through prior exercise is related to the magnitude of this effect [7, 8]. Mestek et al. reported, however, that isocaloric sessions before a meal mitigate PPL independently of the intensity of the exercise session [9]. By contrast, other studies showed that the magnitude of PPL was influenced by prior exercise intensity [5, 10]. Thus, the effects of the intensity of the exercise undertaken on postprandial response remain contentious.

With regard to postprandial endothelial function, evidence has shown that a single bout of exercise prior to HFM consumption improves fasting and postprandial endothelial function compared with a resting control condition [11–13]. Accordingly, it has been reported that acute moderate- and high-intensity exercise has transient benefits for macrovascular endothelial function in both fasting and postprandial states, and that these effects may be due to the improvement in antioxidant status [14, 15]. Nevertheless, the limited prior studies carried out to investigate the effects of exercise intensity have produced inconsistent findings [15–17].

The aforementioned studies investigating the protective effects of exercise performed a few hours before consumption of an HFM on postprandial metabolism and endothelial function have focused on the acute effects of exercise. Thus, although a 12-week training program has been established as a protocol to assess the chronic effects of exercise [18], the potential impact on postprandial metabolism and vascular function after HFM have not been previously investigated. A recent narrative review summarized the current literature on the possible contributions of medium- to long-term physical training to the reduction of the postprandial response, concluding that the data are inconclusive [18]. Interestingly, a recent systematic review and meta-analysis on cardio-metabolic health showed that performing even a short period (∼4 min) of high-intensity exercise has greater benefits than moderate-intensity exercise in terms of cardiometabolic risk factors [19].

Considering that most adults do not meet the public health recommendations of at least 150 min per week of moderate-intensity exercise and also that habitual physical activity declines during middle age [20], it is of special interest to identify how much high-intensity exercise is needed to optimize vascular function in adulthood.

Thus, we Given the above, we hypothesized that medium-term exercise could attenuate the postprandial decrement in metabolism and endothelial function and that this effect would differ according to exercise intensity. On this basis, we aimed to determine the effects of a 12-week high-intensity training (HIT) or a moderate continuous training (MCT) program on postprandial metabolism and vascular function and arterial stiffness after HFM ingestion in healthy, inactive Latin-American adults.

## Methods

### Study design and setting

Details of the study design and methods of the primary HIT-Heart Study trial have been described elsewhere (ClinicalTrials.gov ID: NCT02738385; April 14th, 2016) [21]. The study was performed in accordance with the Declaration of Helsinki (2000) and was approved by the local office for Medical Research Ethics Committee of The University of Santo Tomás, Colombia (ID 27–0500-2015). Postprandial biochemical and vascular function responses were assessed at baseline and over 12 weeks of training. We have previously provided an overview of the methods as per the Consolidated Standards of Reporting Trials (CONSORT) checklist [22].

### Participants

Participants (*n* = 20) were recruited at the University of Rosario (Bogota, Colombia) from February 2015 to May 2016. Subjects were eligible to participate if they were located in the metropolitan region, with available time (1 h per day) to support the trial. Inclusion criteria were individuals aged 18–45 years who were inactive (< 150 min·wk.^− 1^ of moderate-intensity activity or 75 min·wk.^− 1^ of vigorous-intensity activity), had a body mass index (BMI) ≥18 and ≤ 30 kg/m^2^ and identified as being willing and having almost immediate availability. Individuals with a history of any medical condition identified by the American Heart Association as an absolute contraindication to exercise testing were excluded from the study [23]. Furthermore, individuals were also excluded if they presented any of the following: systemic infections, weight loss or gain of > 10% of body weight in the past 6 months for any reason, currently taking medication that suppresses or stimulates appetite, uncontrolled hypertension (systolic blood pressure 160 mmHg or diastolic blood pressure 95 mmHg), gastrointestinal disease (including self-reported chronic hepatitis or cirrhosis, any episode of alcoholic hepatitis or alcoholic pancreatitis within the past year, inflammatory bowel disease requiring treatment in the past year, recent or significant abdominal surgery e.g., gastrectomy), asthma, diagnosed diabetes (type 1 or 2), fasting impaired glucose tolerance (blood glucose ≥118 mg·dl^− 1^), use of any prescribed drugs, any active use of illegal or illicit drugs, or inability to participate because of a physical impairment. In addition, two exercise physiologists tested whether subjects had alterations in ventricular function and/or cardiomyopathy, measured by standard 12-lead electrocardiography (ECG) at rest and every 3 min during a maximum treadmill exercise test. All subjects remained under usual medical care and clinical follow-up (i.e., regular appointments with a physician) throughout the protocol. All participants provided written informed consent before participating in the study.

### Blinding and randomization

The coordinating Research Center for Physical Activity Measurement (CEMA) in Bogotá randomized the procedures with software using randomly permuted blocks. Group allocation was conducted via an online system in which the details of eligible participants were entered to obtain group assignments (i.e., 3:2 or 2:3). Assessors were blinded to study group assignments.

### Interventions

After inclusion, patients performed a maximal cardiopulmonary exercise test on a maximum treadmill exercise test (Precor TRM 885, Precor Corp., Rome, Italy) following the modified Balke protocol [24]. Physiological parameters (maximal O_2_ consumption [VO_2_], heart rate [HR] and Borg ratings) from the test were used to establish the exercise intensity. Based on averaged maximum HR (HRmax) and VO_2_peak, the participants were classified according to normative values, referenced to age and sex. MCT and HIT interventions lasted 12 weeks, with 3 sessions per week consisting of fast walking or running on a treadmill with the deck inclined to reach the desired intensity. HR was recorded each session using an HR monitor (Polar Pacer, Polar Electro, Kempele, Finland). In addition, rating of perceived exertion (RPE) was also measured in each exercise session.

#### Moderate continuous training (MCT) group

Each preparatory period started with an exercise dose of 6 kcal·kg^− 1^·week^− 1^, which was increased progressively by 2 kcal·kg^− 1^·week^− 1^ until week 4 and was then maintained at 12 kcal·kg^− 1^·week^− 1^ for weeks 5 to 12, which was equivalent to 300 kcal of energy expended by the end of the training and cool-down (3 min) periods with total exercise time ranging from 45 to 55 min. Exercise training sessions were designed to elicit a response in the acceptable moderate-to-vigorous range, that is, 60–75% of heart rate reserve (HRR), and were adjusted according to ratings on the Borg scale [25].. During the supervised intervention, HR was recorded using an HR monitor (Polar Pacer) to ensure compliance with the exercise stimulus at the predetermined target HR zone.

#### High-intensity training (HIT) group

We calculated training energy expenditures according to participants’ age ranges and set the target energy expenditures to meet the consensus public health recommendations from the Cardiometabolic HIT-RT Study [25]. Each preparatory period started with an exercise dose of 6 kcal·kg^− 1^·week^− 1^, which was increased progressively by 2 kcal·kg^− 1^·week^− 1^ until week 4 and was then maintained at 12 kcal·kg^− 1^·week^− 1^ for weeks 5 to 12. The overall goal for the HIT group was to perform exercise sessions in 4 × 4-min intervals at 85–95% of HRR (with the target zone maintained for at least 2 min), interspersed with a 4-min recovery period at 75–85% of HRR. During each exercise session, participants adhered to the 12 kcal·kg^− 1^·week^− 1^ energy expenditure format, which was equivalent to 300 kcal of energy expended by the end of the training and cool-down (3 min) periods, with total exercise time ranging from 32 to 45 min. During the supervised intervention, HR and Borg ratings were measured as described for the MCT group.

Both groups were instructed to refrain from exercise training and to avoid changing their physical activity levels outside the study. All participants reported adhering to these instructions. Although diet was not controlled, participants met with the study dietician for nutrition assessment and counseling at baseline, and an individualized iso-energetic nutrition intervention plan was developed from the baseline food intake assessment according to participant preferences. This plan was standardized at 1300–1500 kcal·day^− 1^ (50–55% carbohydrates, 30–35% total fat, < 7% saturated fat and 15–22% protein), distributed across 3–4 meals per day.

### Data collection and outcome measures

#### Experimental procedure

**Fig. 1:**
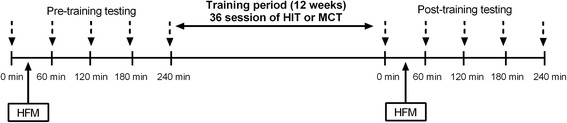
Schedule of experimental events for each subject. HIT, high-intensity interval training; MCT, moderate-intensity continuous training; HFM, high-fat meal. Discontinuous arrows represent capillary blood samples and assessment of endothelial function

All measurements were performed at baseline and at the 12-week follow-up by personnel who were blinded to the treatment allocation. To control for confounding variables, we instructed the subjects to: i) fast for 10–12 h, ii) abstain from exercise for 24 h, iii) abstain from caffeine, tobacco, and vitamin supplements for 12 h, and iv) be awake between 0600 and 0700 h, all prior to each testing session. The HFM, which has been previously reported [26], consisted of a breakfast containing 1049 cal: 79 g of fat, 31 g of saturated fat, 4.5 g of trans fat, 666 mg of cholesterol, 69 g of carbohydrates, 31 g of protein, and 2.22 mg of sodium, adjusted by individual body weight. The effects of the HFM were measured in a fasted state and 60, 120, 180, and 240 min postprandially. Figure 1 represents the schedule of experimental events for each subject.

#### Biochemical parameters

Blood was drawn from an antecubital vein. The biochemical profile included plasma total cholesterol, high-density lipoprotein cholesterol (HDL-c), triglycerides and glucose (measured by enzymatic colorimetric methods). Inter-assay reproducibility (coefficients of variation) was determined via ten replicate analyses of five plasma pools over 15 days and was shown to be 2.0, 3.2, 2.6 and 1.5% for total cholesterol, HDL-c, triglycerides and serum glucose, respectively. All determinations were analyzed in serum using a Cardiocheck® and A1CNow + ® system.

#### Vascular function and arterial stiffness measures

All subjects were tested at the same time of day and after consumption of a low nitrate diet for 48 h. Vascular function and arterial stiffness, as measured by flow-mediated vasodilation (FMD), aortic pulse wave velocity (PWV) and the augmentation index (AIx) were measured. FMD was measured as described in previous studies from our group in the Colombian population [26] using the guidelines reported by Atkinson et al. [27]. The same operator performed all Doppler ultrasound (Mindray M-9® DS USA; Mahwah, NJ) examinations using a 7.5-MHz linear array probe. Ultrasound imaging of the brachial artery was performed with the subjects in the supine position after 15 min of rest, with the arm abducted approximately 80° from the body and the forearm supinated. The ultrasound probe was positioned with a 60° insonation angle in a longitudinal plane at a site 1–3 cm proximal to the antecubital fossa to visualize the anterior and posterior lumen-intima interfaces, to measure diameter and central flow velocity (pulsed Doppler). After the baseline images were recorded, a blood pressure cuff, positioned on the arm, was inflated to 200 mmHg for 5 min; to assess FMD, images were acquired continuously for 3 min after cuff deflation, during the reactive hyperemia period. Brachial artery diameter recording was restarted at least 30 s before cuff deflation and continued for 3 min thereafter. The peak artery diameter and the time to reach this peak after cuff deflation were recorded. Images were recorded on a DVD for subsequent measurements by one observer blinded to the study design. FMD was calculated as the percent rise of peak diameter from the preceding baseline diameter and was measured every 1 min after deflation for 3 min. Normalized brachial artery FMD (FMDn) was calculated according to the allometric relationship between the baseline artery diameter and the peak diameter [27]. The intra-session coefficient of variation was ≤1% for the baseline diameter. Reliability was estimated by intra-class correlation coefficients based on four baseline measurements (*n* = 8 subjects), yielding values of 0.91 for baseline diameter and 0.83 for FMD (our own data). The technical error of measurement was 1.23% for baseline diameter, 1.77% for maximum diameter and 20% for %FMD.

The PWV was measured by analyzing the oscillometric pressure curves registered from the upper arm. Patient data and the measured distance between the jugulum and the symphysis were registered in an arteriographic computer program (Arteriograph Software v.1.9.9.2; TensioMed, Budapest, Hungary). A tape measure was used to measure the distance between the jugulum and the symphysis, the aortic distance. The cuff was placed on the patient’s upper arm and connected to the device. The algorithm measuring blood pressure in the arteriography device has been previously validated [28]. PWV was calculated as the jugulum-to-symphysis distance (m) divided by one-half of the return time (return time/2) (s). For PWV measurements, the two recordings with the lowest standard deviations were chosen. The standard deviation was calculated on the basis of all heartbeats during a period of 8 s.

The AIx was calculated as the ratio of the difference between the systolic peaks of the first pulse [1] and second pulse [2] relative to the central pulse pressure, expressed as a percentage [(pulse 2 - pulse 1/central pulse pressure) × 100]. Thus, it provides the brachial/aortic AIx without applying a transfer function. The R value, used as an estimate of the measurement errors for the repeated measurements between two sessions, was low for the arteriograph (1.18 m·s^− 1^).

### Statistical analysis

To retain the data of all randomly allocated participants, we performed an intention-to-treat analysis (all randomly assigned patients). The Shapiro-Wilk test was used to verify data distribution normality. Once it was confirmed that the sample data satisfied the normality assumption, statistical analyses relevant to our main research interests were conducted. T-tests for continuous variables and chi-squared tests for categorical variables were used to investigate any possible differences in baseline characteristics between the two conditions (HIT and MCT). We used a generalized linear model to analyze the influence of the different training protocols on biochemical and vascular function outcomes with repeated measures [2 (group) × 2 (test time)]. The area under the curve (AUC), expressed in arbitrary units (au) via the trapezoidal method, was calculated and used to analyze the response to the training protocols. The effect of training on AUC measures was analyzed by two-way analysis of variance. Significant differences in AUC from 0 to 240 min after the HFM following 12 weeks of HIT or MCT were analyzed using two-way analysis of variance. A criterion alpha level of *P* ≤ 0.05 was used to determine statistical significance. All data are reported as the mean ± standard deviation. Statistical analyses were conducted using PASW Statistics 17 for Windows (SPSS, Inc., Chicago, IL).

## Results

### Study participants

Additional file 1: Figure S1 (Supplemental Digital Content) shows the flowchart of this randomized clinical trial. A total of 28 physically inactive subjects were assessed for eligibility, of which seven were excluded for not meeting the inclusion criteria. Of the 21 participants who started the study, 20 finished and one participant in the MCT group withdrew for reasons unrelated to the study (lack of time due to work schedule). Ten participants were randomly allocated to the MCT group, and 11 were randomly allocated to the HIT group.

The demographic features of the HIT and MCT groups, as well as their biochemical and endothelial function variables in the fasted state at baseline, are outlined in Table 1. No statistically significant differences (*P* > 0.05) in baseline characteristics between the exercise training protocols were found (t-test), confirming that participants in both groups began the trial under similar conditions.

**Table 1:** Demographic, biochemical and endothelial function variables in the fasted state across HIT and MCT groups at baseline

Characteristics	HIT (*n* = 11)	MCT (*n* = 9)	*P* value
Sex, n (%)			
Male	8 (40.0)	5 (55.6)	0.898
Female	3 (60.0)	4 (44.4)	0.916
Age, mean (sd), y	31.8 (7.8)	31.4 (6.4)	0.928
Biochemical parameters, mean (SD)			
Total cholesterol (mg/dL)	159.4 (47.4)	170.1 (41.8)	0.301
High-density lipoprotein (mg/dL)	46.9 (9.6)	43.0 (14.1)	0.236
Triglycerides (mg/dL)	100.4 (36.8)	134.1 (82.2)	0.118
Glucose (mg/dL)	78.3 (5.6)	82.3 (13.7)	0.190
Vascular function parameters, mean (SD)			
D_base_, mm	3.0 (0.6)	2.7 (0.4)	0.157
FMD, %	7.2 (3.3)	7.3 (5.6)	0.487
*D*_peak_ mm	3.2 (0.5)	3.0 (0.5)	0.140
*D*_diff_	0.2 (0.5)	0.3 (0.4)	0.496
FMDn, %	6.5 (2.9)	7.4 (5.7)	0.433
PWV, m·s^−1^	6.7 (0.8)	7.1 (1.2)	0.204
AIx (aortic), %	41.7 (10.4)	24.5 (32.7)	0.148
AIx (brachial), %	16.5 (5.2)	25.1 (16.5)	0.152
Pulse Pressure (mmHg)	45.3 (5.2)	44.6 (4.1)	0.931
Central systolic blood pressure (mmHg)	99.6 (43.7)	108.0 (5.8)	0.493
Pulmonary artery occlusion pressure (mmHg)	40.6 (6.8)	35.6 (3.9)	0.087
Systolic blood pressure (mmHg)	116.2 (6.5)	116.8 (5.1)	0.184
Diastolic blood pressure (mmHg)	71.0 (8.7)	72.3 (7.0)	0.278
Mean blood pressure (mmHg)	86.0 (7.6)	87.3 (6.0)	0.482

*HIT* 4 × 4-min high -intensity interval training, *MCT* moderate-intensity continuous training, *D* diameter, *FMD* flow-mediated vasodilation, *nFMD* normalized flow-mediated vasodilation, *PWV* pulse wave velocity, *AIx* augmentation index

### Biochemistry and endothelial response

Postprandial biochemical and endothelial function responses with summary measures of these responses after 12 weeks of HIT or MCT are shown in Table 2. A significant difference in glucose between 0 min (fasted state) and 120 min postprandially in the HIT group was found (*P* = 0.035). Likewise, %FMD was significantly different between the fasted state and 60 min after HFM in the HIT group (*P* = 0.042).

**Table 2:** Intent-to-treat analysis of the effect of 12 weeks of HIT or MCT on postprandial lipemia biochemical and vascular function response after HFM ingestion

	HIT					MCT				
	0 min	60 min	120 min	180 min	240 min	0 min	60 min	120 min	180 min	240 min
Biochemical parameters										
Total cholesterol (mg/dL)	151.3 (21.0)	159.0 (28.1)	159.0 (30.2)	163.2 (30.0)	164.8 (20.2)	153.1 (29.09)	158.6 (25.1)	165.1(25.1)	166.8 (25.0)	167.1 (29.4)
High-density lipoprotein (mg/dL)	46.0 (14.1)	47.3 (14.6)	46.1 (12.7)	48.1(16.1)	48.7(15.9)	42.1 (9.5)	44.2 (11.6)	42.5 (12.2)	41.6 (12.6)	40.5 (10.9)
Triglycerides (mg/dL)	108.1 (35.8)	118.7 (41.7)	142.1 (53.7)	177.2 (91.5)	171.7 (84.5)	117.7 (33.1)	132.4 (31.1)	196.8 (56.1)	222.7 (68.2)	222.5 (91.2)
Glucose (mg/dL)	76.4 (11.0)	78.8 (12.9)	88.5 (8.8)^b^	84.3 (7.8)	86.3 (5.0	85.9 (6.3)	93.6 (12.4)	94.2 (7.8)	93.3 (10.3)	89.5 (10.7)
Vascular function parameters										
D_base_, mm	2.7 (0.4)	2.8 (0.3)	2.7 (0.3)	2.7 (0.4)	2.8 (0.3)	3.1 (0.5)	3.2 (0.4)	3.2 (0.5)	3.3 (0.5)	3.4 (0.4)
FMD, %	13.4 (4.6)	6.3 (7.3)^a^	12.3 (5.3)	12.0 (5.8)	10.4 (4.6)	9.4 (4.0)	6.1 (3.9)	7.5 (3.1)	7.3 (5.7)	6.7 (4.5)
*D*_peak_ mm	3.0 (0.3)	2.9 (0.3)	3.1 (0.3)	3.0 (0.3)	3.1 (0.4)	3.4 (0.5)	3.4 (0.5)	3.4 (0.4)	3.6 (0.5)	3.6 (0.4)
*D*_diff_	0.3 (0.2)	0.1 (0.1)	0.4 (0.3)	0.3 (0.2)	0.3 (0.2)	0.3 (0.2)	0.2 (0.1)	0.2 (0.1)	0.3 (0.2)	0.2 (0.1)
nFMD, %	13.5 (6.3)	13.4 (4.6)	11.7 (6.1)	11.8 (7.3)	10.1 (5.1)	8.1 (4.1)	9.4 (4.0)	6.5 (3.2)	6.3 (5.2)	5.5 (4.2)
PWV, m·s^−1^	6.6 (1.5)	7.0 (1.6)	6.6 (2.4)	6.7 (1.2)	6.8 (1.0)	6.7 (0.9)	6.5 (1.0)	6.7 (1.0)	6.5 (1.0)	6.7 (1.0)
AIx (aortic), %	26.3 (14.6)	14.7 (9.4)	15.9 (12.1)	18.3 (12.5)	19.1 (9.2)	38.7 (69.0)	7.6 (4.2)	3.6 (16.0)	9.5 (4.1)	11.2 (7.0)
AIx (brachial), %	−22.3 (28.9)	−45.2 (18.7)	−42.7 (24.0)	−38.1 (24.8)	−36.5 (18.2)	−41.7 (16.5)	−59.3 (8.5)	−34.6 (45.0)	−55.5 (8.2)	−52.0 (13.8)
PP(mmHg)	45.5 (7.7)	49.8 (7.3)	45.9 (8.5)	48.5 (10.4)	47.4 (7.4)	45.1 (7.6)	46.7 (6.4)	51.0 (8.4)	46.0 (6.6)	48.8 (7.2)
SBPao (mmHg)	107.6 (14.2)	102.4 (7.7)	102.6 (12.9)	105.8 (10.2)	106.3 (9.6)	104.3 (7.3)	103.6 (8.4)	104.5 (6.1)	105.0 (8.4)	109.3 (9.6)
PPao (mmHg)	40.6 (6.6)	39.0 (3.4)	36.9 (7.1)	39.7 (6.3)	39.3 (5.1)	30.7 (12.9)	33.8 (5.3)	38.0 (6.9)	35.0 (6.2)	36.8 (6.1)
Systolic blood pressure (mmHg)	112.5 (9.1)	113.2 (6.4)	111.5 (8.4)	114.6 (7.2)	114.3 (6.6)	113.0 (7.6)	116.4 (9.1)	118.6 (6.1)	117.0 (8.1)	121.2 (10.8)
Diastolic blood pressure (mmHg)	67.0 (10.3)	63.4 (8.8)	65.6 (11.4)	66.1 (9.1)	67.0 (8.0)	67.8 (9.4)	69.7 (6.2)	66.5 (6.6)	71.0 (11.7)	72.4 (6.8)
Mean blood pressure (mmHg)	82.0 (9.2)	80.0 (7.4)	80.8 (9.7)	82.2 (7.0)	82.9 (6.7)	82.8 (7.8)	85.3 (6.8)	83.7 (5.2)	86.2 (10.2)	88.7 (7.7)

Values are participant characteristics at baseline, mean (SD). *HIT*4 × 4-min high-intensity interval training, *MCT* moderate-intensity continuous training, *D* diameter, *FMD* flow-mediated vasodilation, *FMDn*, normalized flow-mediated vasodilation, *PWV* pPulse wave velocity, *AIx*, augmentation index, *PP* pulse pressure, *SBPao* central systolic blood pressure. *PPao*, pulmonary artery occlusion pressure. Whole group repeated measures ANOVA, a = 0 min vs 60 min; b = 0 min 120 min

**Fig. 2:**
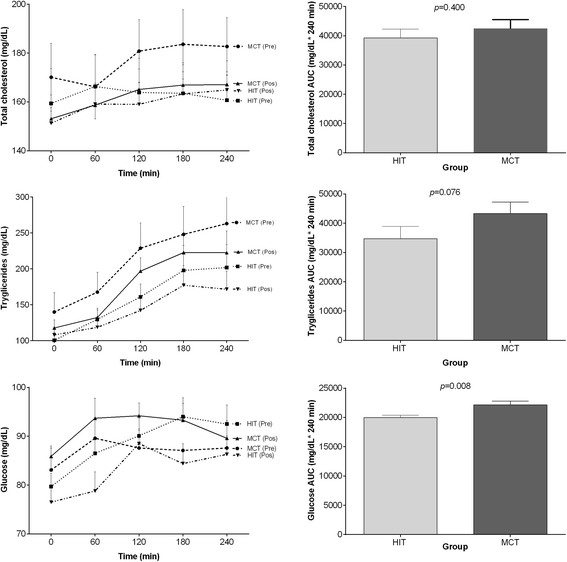
Total cholesterol, triglycerides (TG) and glucose responses to PPL (left) and incremental AUC (right) after 12 weeks of HIT and MCT. Deviations from fasting were analyzed by repeated-measures ANOVA. Differences between total cholesterol, TG and glucose AUC_(0–240)_ after 12 weeks of HIT of MCT were analyzed by two -way analysis of variance

Figure 2 shows the effects of HIT and MCT on total cholesterol, triglycerides and glucose postprandial responses, with summary measures of these responses. The total cholesterol response expressed as AUC_(0–240)_ was lower following HIT than following MCT, but no significant differences were observed (8%, *P* > 0.05). Similarly, triglycerides AUC_(0–240)_ was also lower following HIT than following MCT, with a trend toward significance (24%, *P* = 0.076). AUC_(0–240)_ for the glucose response was significantly lower following HIT than MCT (10%, *P* = 0.008).

**Fig. 3:**
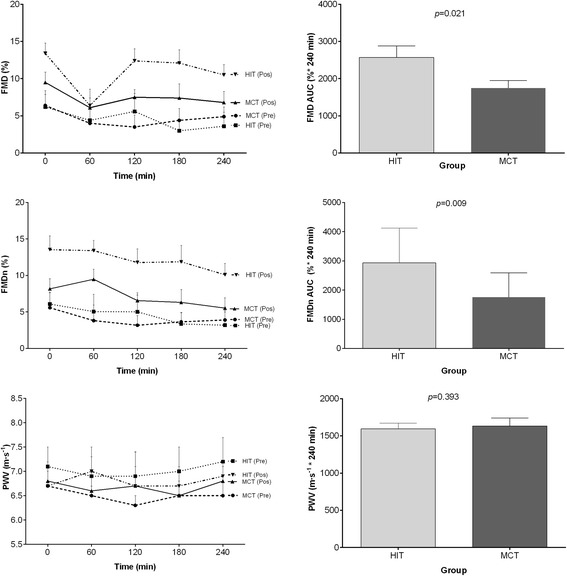
FMD (%), FMDn (%) and PWV responses to PPL (left) and incremental AUC (right) after 12 weeks of HIT and MCT**.** Deviations from fasting were analyzed by repeated-measures ANOVA. Differences between FMD (%), FMDn (%) and PWV AUC_(0–240)_ after 12 weeks of HIT of MCT were analyzed by two-way analysis of variance

Figure 3 shows the effects of HIT and MCT on FMD, nFMD and PWV postprandial responses, with summary measures of these responses. FMD and nFMD AUC_(0–240)_ were significantly higher following HIT than following MCT (46.9%, *P* = 0.021 and 67.3%, *P* = 0.009, respectively). PWV AUC _(0–240)_ did not differ between HIT and MCT (2.3%, P > 0.05).

No adverse events were reported during this study. As we have previously reported, the average exercise-training days and total exercise time during the program were 35.5 days (SD 1.3) and 1100 min (SD 258) in the MCT group and 35.4 days (SD 0.9) and 1031 min (SD 147) in the HIT group (*P* = 0.043) [21].

## Discussion

The aim of this study was to investigate the effects of chronic MCT and HIT on postprandial lipemia and vascular function and arterial stiffness after HFM consumption in inactive adults. The main finding of this study is that a 12-week regimen of HIT reduces glucose concentrations and exerts greater post-HFM endothelial function than MCT, supporting the idea that the effect of chronic exercise on postprandial response is dependent on exercise intensity [29].

Recent evidence has shown that acute exercise increases FMD following HFM consumption [12, 30]. To the best of our knowledge, the present study is the first to demonstrate that medium-term exercise training prevents the decline in FMD induced by PPL, supporting the protective effect of regular exercise on vascular function. This is clinically relevant since endothelial function is an independent risk factor of CVD [3]. Interestingly, the higher AUC values of FMD in the HIT group after HFM (*P* = 0.009) indicates that this regimen may provide major vascular benefits in inactive adults.

In agreement with our results, previous studies focusing on the effect of acute exercise demonstrated that FMD remained greater after HFM consumption following a single bout of HIT as compared with MCT [15, 31]. However, although it is of special interest to calculate incremental AUC values drawn from hourly measurements up to 4 h using the trapezoid rule [12, 32], most previous studies have not included these data. In contrast to our present findings, a recent study conducted in 11 physically active young men reported that FMD response did not differ between the two conditions [16]. The differences in training status between this study population (physically active) and our study cohort (inactive) might explain the inconsistent findings, since it has been shown that FMD responses after HFM consumption may differ between active and inactive subjects [33]. Thus, based on our results and previous research, it can be hypothesized that exercise attenuates the negative effects of HFM consumption on endothelial cell function in an inactive population. Further studies investigating the mechano-sensory mechanisms contributing to the effect of exercise on vascular function as well as possible interactions among molecular pathways are required [34].

The mechanism by which chronic exercise training can modulate postprandial endothelial function is unclear. Regular exercise has been proposed to decrease PPL and therefore reduce postprandial oxidative stress by maintaining low lipoprotein levels [35]. An alternative mechanistic explanation is that regular exercise might increase antioxidant capacity, leading to maintenance of endothelial function [15]. Indeed, a substantial increase in exercise intensity has been linked to greater protection of vascular function against oxidative stress, supporting the possibility that HIT might trigger larger vascular effects at the cellular and molecular levels [15]. Likewise, exercise might exert a positive effect on endothelial function by stimulating the production and bioavailability of NO, as physical activity induces the activity of endothelial NO synthase (eNOS), increases the capacity of the cellular antioxidant system and diminishes the formation of reactive oxygen species (ROS) [36]. In addition, it has been demonstrated that a single session of exercise increased circulating and intramuscular free radical levels [37], which may lead to inactivation of NO with consequences for endothelium-mediated vasodilation [38]. It seems that acute exercise mediates the oxidant-antioxidant balance in favor of antioxidants, resulting in the maintenance of vascular function, and a similar effect is observed from the co-ingestion of antioxidants [39]. Thus, it is tempting to speculate that the effects of different intensities of exercise on postprandial FMD are related to changes in antioxidant status.

We also found that medium-term HIT decreased the glucose response over the postprandial observation period by 10% compared with MCT (*P* = 0.008), indicating that the 7magnitude of postprandial glucose response was dependent on exercise intensity. This result contrasts with previous reports that failed to find differences between the two training protocols regarding postprandial glucose levels [10, 15, 16]. However, it should be noted that these studies only examined the effect of postprandial glucose level after acute exercise. Thus, it is possible that only medium or long-term training has a significant effect on postprandial glucose response.

We found similar total cholesterol, HDL-c and triglyceride responses after HFM consumption in both training regimens, suggesting that medium-term exercise training might not play an important role in the postprandial decrement in lipid responses. In previous studies focusing on acute exercise, significant differences between HIT and MCT were found for triglycerides, but not for total cholesterol or HDL levels [10, 15, 16]. In this context, results from preliminary studies have suggested that the positive effect of exercise training on PPL might be short lived, demonstrating variations in the effect sizes for exercise training performed within 24 h prior to HFM ingestion and for exercise training performed more than 24 h pre-prandial [8, 40]. Thus, we hypothesize that postprandial triglyceride response might be short lived, showing a relevant effect only after acute exercise.

### Study limitations

This study has some limitations. Due to the high sensitivity of endothelium to nutritional changes, it would be ideal to administer isocaloric meals to participants at least three days before the measurement of endothelial function. In this study, although diet was not controlled, a dietician provided an individualized iso-energetic nutrition intervention plan. Second, since endothelial function is well known to be affected by age and training status and our study cohort comprised healthy, inactive mature adults, our findings may not be generalizable to other populations with different characteristics. A final possible limitation is that we did not examine other factors such as antioxidant status, NO, IL-6 and TNF-α levels that might ameliorate postprandial response, and this should be studied in future research.

The main strength of our study is that, to our knowledge, this is the first randomized clinical trial on the effect of exercise-training intensity on biochemical parameters and endothelial functional responses to HFM consumption in inactive adults from the Latin-American population. In addition, we provide measurements of these PPL responses at multiple time points to better describe their time course after chronic exercise.

## Conclusion

In summary, the novel finding of this study was that medium-term supervised physical training may mitigate endothelial dysfunction and glucose response induced by PPL. Exercise intensity seems to play an important role in these protective effects, suggesting that HIT might be the more effective in reducing postprandial glucose levels and attenuating vascular impairments. Therefore, medium-term HIT is an effective strategy to reduce CVD.

## Additional file

Additional file 1: **Figure S1.** CONSORT guidelines flow diagram for enrolment and randomization. (TIFF 1515 kb)
