# Supplementary figures and images for: Exercise and postprandial lipemia: effects on vascular health in inactive adults
Source: Lipids Health Dis. 2018 Apr 3;17:69. doi: 10.1186/s12944-018-0719-3 (PMC5883528; doi:10.1186/s12944-018-0719-3)

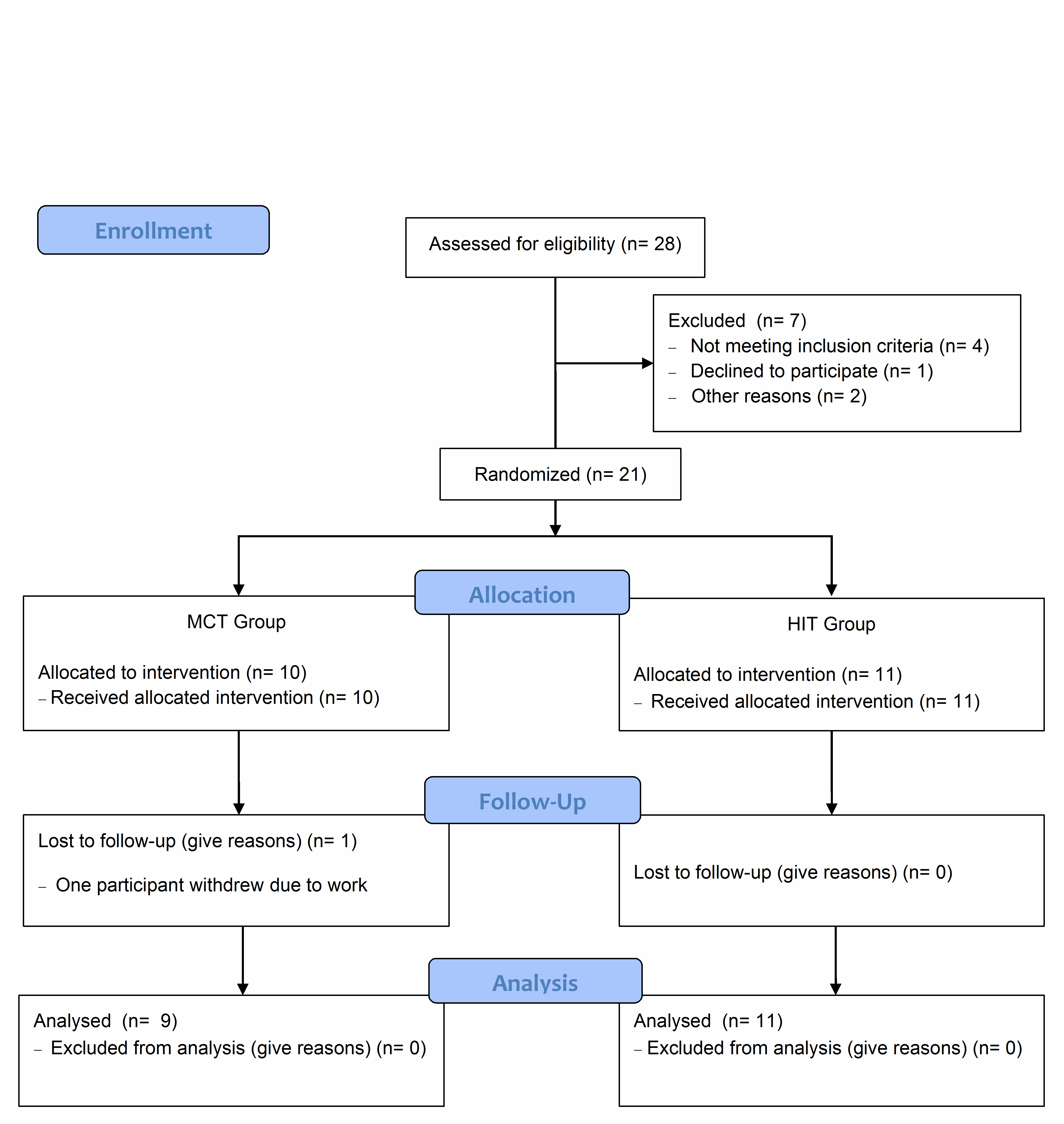

Supplement: Supplementary file 1 — Figure S1. CONSORT guidelines flow diagram for enrolment and randomization. (TIFF 1515 kb) [file 12944_2018_719_MOESM1_ESM.tif]
